# Association of variants in *AGTR1*, *ACE*, *MTHFR* genes with microalbuminuria and risk factors for the onset of diabetic nephropathy in adolescents with type 1 diabetes in the population of Serbia

**DOI:** 10.1371/journal.pone.0312489

**Published:** 2024-10-24

**Authors:** Smiljka Kovacevic, Vera Zdravkovic, Jelena Blagojevic, Stefan Djordjevic, Jelena Miolski, Vladimir Gasic, Marina Jelovac, Milena Ugrin, Sonja Pavlovic, Maja Jesic

**Affiliations:** 1 Endocrinology Department, University Children’s Hospital, Belgrade, Serbia; 2 Faculty of Medicine, University of Belgrade, Belgrade, Serbia; 3 Rheumatology Department, University Children’s Hospital, Belgrade, Serbia; 4 Department of Pediatrics, General Hospital Stefan Visoki, Smederevska Palanka, Serbia; 5 Laboratory for Molecular Biomedicine, Institute of Molecular Genetics and Genetic Engineering, University of Belgrade, Belgrade, Serbia; PearResearch / Government Doon Medical College, INDIA

## Abstract

**Introduction:**

Genetic studies may provide valuable information about patients who are at high risk of developing diabetes nephropathy. Before the appearance of albuminuria, there are genetic mutations that can predispose the development of kidney disease.

**Material and methods:**

The study included 130 adolescents with type 1 diabetes. Patients were divided into two groups according to the presence of microalbuminuria. This study was performed to examine clinical and laboratory differences between adolescents with type 1 diabetes with and without microalbuminuria and the distribution of the ACE, AGTR1, and MTHFR gene polymorphisms.

**Results:**

The mean microalbuminuria in the first group 6.41±7.35 significantly differs from the second group 0.82±0.48 (p<0.001). HbA1c, 24-hour proteinuria, and day-time systolic blood pressure were significantly higher in the MA group (p<0.05). Smaller systolic blood pressure percentage nocturnal decline was observed in the microalbuminuric group (p 0.030). The frequencies of the ACE DD, ID, and II genotypes were 12.5%, 50.0%, and 37.5%, respectively, among T1D patients with MA, and 19.3%, 56.1%, 24.6%, in the control group without MA (P = .510). The frequencies of the AGTR1 AA, AC, and CC genotypes were 62.5%, 25.0%, and 12.5% among TID patients with MA, and 49.1%, 43.9%, 8.0%, in the group without MA (p 0.326). The frequencies of the MTHFR CC, CT and TT genotypes were 37.5%, 50.0%, 12.5% among TID patients with MA, and 37.7%, 45.6%, 16.7% in the group without MA (p 0.901).

**Conclusion:**

Our data suggest that common variants in the *AGTR1*, *ACE*, and *MTHFR* genes are not strongly associated with diabetic nephropathy in our patients with type 1 diabetes.

## Introduction

Mortality of patients with diabetes mellitus type 1 (T1D) is mostly related to developing nephropathy as a major microvascular complication [1]. The rising trend of diabetic nephropathy (DN) indicates the increasing number of patients with end-stage renal disease (ESRD) due to diabetes [2]. Microalbuminuria (MA) is used as an early indicator of clinically detectable diabetic nephropathy (DN). The prevalence of MA is 7–20% of young adults and children with T1D [3, 4]. However, the predictive value of microalbuminuria is limited. It is shown that normal-range albuminuria does not exclude nephropathy in diabetic children [5]. Some patients without microalbuminuria develop advanced renal pathological changes, meaning that microalbuminuria may not be an optimal marker for the early detection of diabetic kidney disease [5]. In those patients incidence of ESRD was 7.8 times higher than that in normoalbuminuric individuals without diabetes [6].

On the other hand genetic studies provide valuable information about patients who are at high risk of developing DN. Before the appearance of albuminuria, there are genetic mutations that can predispose the development of kidney disease. By identifying these factors, we can slow down the progression of kidney damage in predisposed individuals. One of the candidate genes that may predispose to developing DN are angiotensin-converting enzyme (ACE), angiotensin II receptor type 1 (AGTR1), and methylene-tetrahydrofolate reductase (MTHFR).

An activated intrarenal renin–angiotensin system has been implicated as a crucial player in the pathogenesis of DN, as it plays a role in glucose metabolism, regulating blood pressure and fluid homeostasis. ACE, angiotensinogen (Atg) and AGTR1 are part of RAS. ACE indel polymorphism accounts for almost half of the variability in circulating ACE levels [7]. The ACE I/D polymorphism regulates ACE activity and has a role in the development and progress of DN [8]. The I/D ACE gene polymorphism is an independent factor influencing the development of cardiovascular complications and the development of DN in patients with DM [8].

The gene for AGTR1 regulates the secretion of aldosterone and has an impact on blood pressure control and the work of the cardiovascular system. Some effects of the ACE indel and the AGTR1 act in synergy to increase the risk of cardiovascular diseases (CVD) [9].

Genetic variants involved in the epigenetic mechanism of DN development are polymorphisms of the MTHFR gene. In patients with DM low folate and elevated homocysteine are connected with endothelial dysfunction and microvascular complications in DM [10, 11]. The polymorphism in MTHFR 677C -T is associated with elevated homocysteine and microvascular complications in patients with DM [12].

The aim of this study was to examine clinical and laboratory differences between adolescents with T1D with and without MA. Secondly, we examined the distribution of the ACE, AGTR1, and MTHFR gene polymorphisms in patients with T1D divided into two groups depending on the presence or absence of MA. Polymorphisms of three studied genes were correlated with the clinical characteristics and laboratory results of our patients.

## Material and methods

### Study design and participants

A cross-sectional, single-center study was performed at the University Children’s Hospital in Belgrade, Serbia. The study was performed between 2020 and 2023. The recruitment period started on December 15^th^, 2020. and ended on June 1^st^, 2023. Participants were 130 adolescents, 52 girls and 78 boys aged between 11–18 years (mean age 14.52±2.34) with a duration of T1D for 2 or more years (mean duration 5.47±3.36). All patients were treated with intensive insulin therapy or an insulin pump. Patients were divided into two groups according to the presence of MA: in the first group were patients with microalbuminuria and in the second group normoalbuminuric patients. Microalbuminuria (mg/L) was determined by turbidimetric method on a Siemens EXL 200 apparatus (Erlanger, Germany). Screening for MA was performed by using the median result of measuring albumin-to-creatinine ratio (ACR) in two or three-morning urine samples in a period of up to 6 months. Persistent MA was confirmed in the patients with the finding of ACR rating 2.5–25 mg/mmol in males and 3.5–25 mg/mmol. All patients had 24-h urine collection. Normoalbuminuria was defined as 24-hour albumin excretion (AER) < 30 mg/24 h and MA as AER > 30 mg– 300 mg. Blood samples consisted of measurements of HbA1c, total cholesterol, triglycerides, high-density lipoprotein (HDL), low-density lipoprotein (LDL), creatinine, cystatin C and were done by routine laboratory methods. Subjects were measured for height and weight and the body mass index (BMI) was calculated.

Glycosylated HbA1c was determined by turbidimetric immunoassay using Siemens Dimension EXL 200 (Erlanger, Germany). Blood pressure was measured by twenty-four-hour ambulatory blood pressure monitoring based on an oscillometric method with the appropriate cuff size.

### DNA isolation and genotyping

Peripheral blood samples were used to isolate using the standardized protocol for DNA extraction using the QIAamp DNA Blood Mini Kit (Qiagen, Hilden, Germany). Afterward, concentrations were measured using the Biospec-nano Shimadzu Spectrophotometer for Life Sciences (Shimadzu, Kyoto, Japan). To genotype the variants ACE rs1799752, MTHFR rs1801133, and AGTR1 rs5186, the respective regions were first amplified using PCR. The ACE rs1799752 variant was genotyped by evaluating the sizes of the amplicons based on the method described [13] with slight modifications. The AGTR1 rs5186 variant was detected using Sanger sequencing [14]. The method is based on amplifying the DNA segment under investigation, then annealing the amplified DNA to an oligonucleotide primer, which is then extended by a DNA polymerase, incorporating both deoxynucleotide triphosphatase molecules and chain-terminating dideoxynucleotide triphosphatase molecules. Afterwards, the collected chromatograph data, which records the type of nucleotide incorporated based on the measured wavelength, is analyzed using sequencing analysis software.

The MTHFR rs1801133 was genotyped using PCR RFLP, using the HinfI restriction enzyme.

### Ethics statement

The study protocol was approved by the Ethics Committee of the University Children’s Hospital in Belgrade (date Nov 26th, 2020). Every participant and parent gave fully informed written consent to participate in the study.

### Statistical analysis

Descriptive and analytical statistical methods were used in data processing. The Shapiro-Wilk test was used to assess the normality of continuous variables’ distribution. Continuous variables were reported as either the mean ± standard deviation (SD) or the median (with minimum-maximum range), based on their normality distribution. Categorical variables were presented as number (percentage). A chi-squared or Fisher’s exact test was used for the analysis of categorical data. The comparison of continuous variables between two groups was conducted using either the independent samples t-test or the Mann-Whitney U test depending on the normality of data distribution. When comparing continuous variables with a normal distribution across three groups, a one-way ANOVA was performed, using Tukey’s method for post hoc analysis. In contrast, the Kruskal-Wallis H test, supplemented by the Mann-Whitney U test for individual group comparisons, was utilized for continuous variables lacking normal distribution across three groups. Statistical significance was set at p < 0.05. Univariate logistic regression analyses were performed for microalbuminuria as an outcome. The multivariable logistic regression with backward Wald as a method was performed to identify factors associated with the presence of microalbuminuria. Variables that were significantly associated with these outcomes at the significance level <0.1 in the univariate logistic regression analysis and based on clinical significance were entered into the multivariable logistic regression model. Odds ratios (OR) with 95% confidence intervals (CI) were computed and the Hosmer–Lemeshow goodness-of-fit test was performed to assess overall model fit. The statistical analyses were performed using SPSS version 23.0 software (SPSS Inc., Chicago, IL, USA).

## Results

Sixteen patients (12.3%) had MA. The mean MA in the first group 6.41±7.35 significantly differs from the second group 0.82±0.48 (p<0.001). In our patients, HbA1c, 24-hour proteinuria, and day-time systolic blood pressure (sBP) were significantly higher in the MA group (p<0.05). A smaller percentage of nocturnal decline was observed in the microalbuminuric group (p 0.030). There was no statistically significant difference in all other blood pressure values. No difference in BP parameters was observed between male and female patients. There were more patients with MA and systolic blood pressure above the 90^th^ percentile (p 0.05). Anthropometric parameters that didn’t differ between MA and non-MA groups are shown in Table 1. The duration of diabetes in years did not affect the differences between the obtained values.

**Table 1:** Clinical characteristics and laboratory results of adolescents with T1D with and without MA.

	All patients	Group 1 (patients with microalbuminuria)	Group 2 (normoalbuminuria)	p-value
n	130	16	114	
Boys, n (%)	72 (55.4)	11 (68.8)	61 (53.5)	0.379
Girls, n (%)	58 (44.6)	5 (31.2)	53 (46.5)	
Age, years	14.52±2.34	15.03±2.28	14.45±2.35	0.352
Duration of diabetes, years	5.47±3.36	6.12±3.66	5.38±3.32	0.406
Body mass, kg	55.21±13,09	53,48±11,16	55.46±13.36	0.547
BMI kg/m2	20.18±3.01	19.82±3.05	20.14±3.02	0.604
Daily dose U/kg body weight	0.80±0.28	0.78±0.24	0.80±0.29	0.766
Family history of CVD, n(%)	26 (20)	3 (18.8)	23 (20.2)	0.894
HbA1c (%)	7.8±1.25	8.86±2.03	7.65±1,03	<0.001
Microalbuminuria	1.51±3.15	6.41±7.35	0.82±0.48	<0.001
Proteinuria 24h urine	0.13±0.15	0.23±0.32	0.12±0.11	0.007
Creatinine clearance (ml/min)	117.52±37.49	113.47±25.41	118.08±38.92	0.668
Cholesterol	4.70±1.02	4.91±1.04	4.67±1.02	0.385
Triglycerides	1.08±1.30	1.30±0.97	1.05±1.34	0.121
HDL	1.77±0.37	1.83±0.50	1.77±0.38	0.563
LDL	2.53±0.89	2.87±1.43	2.49±0.79	0.112
CystatinC	0.77±0.13	0.73±0.12	0.77±0.14	0.272
sBP, mmHg Daytime	111.83±11.24	104.13±23.60	112.95±7.64	0.004
sBP, mmHg Night-time	105.26±7.98	103.6±10.81	105.51±7.52	0.390
dBP, mmHg Daytime	67.54±6.79	65.4±8.08	67.8±56.57	0.192
dBP, mmHg Nigh-time	61.86±7.05	59.93±6.73	62.15±7.08	0.258
% fall sBP	9.49±4.81	6.98±3.6	9.86±4.87	0.030
% fall dBP	11.47±5.48	9.35±5.18	11.78±5.48	0.108

Variables are shown as mean ± SD, n-number of patients

CVD- cardiovascular diseases, sBP- systolic blood pressure, dBP-diastolic blood pressure

On univariate analysis, variables significant for developing MA were: higher HbA1c (p = 0.001), higher day-time sBP (p = 0.004), and smaller sBP percentage nocturnal decline (p = 0.035). Using multivariate logistic regression the variable which remained associated with microalbuminuria was higher HbA1c (odds ratio–OR 2.12;95% confidence interval –95% CI 1.40–3.21; p<0.001).

### Genetic analyses

All patients were genotyped for the ACE, AGTR1, and MTHFR gene polymorphisms. The distribution of genotype frequencies is shown in Table 2. No statistically significant difference among the two groups of patients was found in the genotype distribution for the ACE, AGTR1, and MTHFR gene polymorphisms. There is also no statistical significance in the frequency of genotypes between both sexes.

**Table 2:** Analysis of frequency distribution of ACE, MTHFR, and AGTR1 gene genotypes.

Genotype	Group 1 Microalbuminuria n (%)	Group 2 Normoalbuminuria n (%)	Total	p-value
ACE rs1799752				0.510
D/D	2 (12.5)	22 (19.3)	24 (18.5)	
D/I	8 (50.0)	64 (56.1)	72 (55.4)	
I/I	6 (37.5)	28 (24.6)	34 (26.2)	
MTHFR C677tra1801133				
C/C	6 (37.5)	43 (37.7)	49 (37.7)	0.901
C/T	8 (50)	52 (45.6)	60 (46.2)	
T/T	2 (12.5)	19 (16.7)	21 (16.2)	
AGTR1 rs5186				
A/A	10 (62.5)	56 (49.1)	66 (50.8)	0.326
A/C	4 (25)	50 (43.9)	54 (51.5)	
C/C	2 (12.5)	8 (7.0)	10 (7.7)	

The frequencies of the ACE DD, ID, and II genotypes were 12.5%, 50.0%, and 37.5%, respectively, among T1D patients with MA, and 19.3%, 56.1%, 24.6%, in the control group without MA (P = .510). Patients with the ID genotype had a significantly higher frequency of cardiovascular diseases in the family history (p 0.006). There is a significant difference in creatinine clearance between three different genotypes (p 0.042). Also, patients with the II genotype had a significantly higher creatinine clearance compared to the ID genotype (p 0.049). Patients carrying the DD genotype had increased median arterial pressure (MAP) compared to the II and ID genotypes (p 0.05). In our patients with different genotypes for the ACE gene, there were no significant differences in anthropometric or metabolic parameters.

The frequencies of the AGTR1 AA, AC, and CC genotypes were 62.5%, 25.0%, and 12.5% among TID patients with MA, and 49.1%, 43.9%, 7.0%, in the group without MA (p 0.326). The incidence of different genotypes between MA and non-MA groups had no statistical significance (p 0.326). There is a significant difference in dBP percentage declines between the 3 genotypes of the AGTR1 gene (p 0.017). Patients carrying the AC genotype had statistically significantly smaller dBP percentage declines than patients carrying the AA genotype (p 0.025). There were no significant differences in anthropometric or metabolic parameters.

The frequencies of the MTHFR CC, CT and TT genotypes were 37.5%, 50.0%, 12.5% among TID patients with MA, and 37.7%, 45.6%, 16.7% in the group without MA (p 0.901). The incidence of different genotypes had no statistical significance (p 0.901). There is a statistically significant difference in triglyceride values between 3 different genotypes of the MTHFR gene (p 0.042). Also, patients carrying the CT genotype had higher triglyceride values compared to the TT genotype (p 0.009). There was no statistically significant difference in cholesterol, HDL, or LDL. There is also a statistically significant difference in cystatin C values among 3 different genotypes (p 0.037). Patients carrying the CC genotype had lower cystatin C values compared to the CT genotype (p 0.033). Patients with CC genotype had a higher percentage of nocturnal decline compared to other genotypes (p 0.021).

Our analysis showed a higher prevalence of ID genotype (50%) of the ACE gene, AA genotype AGTR1 gene (62.5%), and CT genotype (50%) of the MTHFR gene in the MA group.

## Discussion

The prevalence of MA was 12.3% but no one had macroalbuminuria. The prevalence of MA was lower than previously reported at 16% on the territory of Serbia [15]. Our patients with MA had significantly higher 24-hour proteinuria, glycosylated HbA1c, day-time sBP, and smaller sBP and dBP percentage nocturnal decline. Unlike other studies, gender was not a risk factor for developing DN in our study. Most microalbuminuric patients were boys (68.8%). The survey from Germany and Austria revealed that male sex was a risk factor for DN progression. On the contrary, other studies from Oxfordshire, Liverpool, UK, and Ireland showed that the development of MA is accelerated in girls [3, 4].

The association of DN with age and duration of diabetes has been shown in many T1D studies, especially that postpubertal years of diabetes contribute more heavily to the risk of developing diabetic nephropathy [4, 16]. In our study that association wasn’t shown probably because 40% of our patients had a duration of diabetes for 3 years or less. The mean duration of diabetes was 5 years so we predict that the prevalence of MA will increase with longer diabetes duration.

As shown in many studies [5, 6, 17], a difference in mean HbA1c was observed between those with and without MA.

Hypertension accelerates the progression of renal disease. Elevated BP is a sign of progressive incipient nephropathy. An elevated diastolic BP confers a risk for the progression of chronic kidney disease in children [4, 18]. Elevated nighttime MAP and diastolic BD in patients with MA leads to poor outcomes [19]. In our study, elevated systolic blood pressure was significantly associated with DN. A similar finding has been shown in a study of the adult T1D population in the territory of Serbia [20].

In this study, a similar distribution of the ACE genotypes was observed in patients with and without MA. In the group with MA, the majority of patients (50%) had an ID genotype of the ACE gene, which is similar to the previous smaller study done on adolescents in the population of Serbia [15]. Age, sex, and duration of diabetes did not differ between patients with II, ID, and DD genotypes. ID genotype was the most frequent in the non-MA group (56.1%) as well as in the total sample of patients (55.4%, 72 patients). Between the two groups of patients with and without MA, there were no differences in the distribution of the ACE ID, DD, II polymorphism (P 0.510), which may be explained by the small number of patients in the group without MA.

People with the DD genotype have higher serum levels of ACE and angiotensin II [21]. Also, the ACE DD genotype is associated with a significantly higher excretion of albumin in the urine and a higher frequency of persistent albuminuria, which leads to the development of DN [8, 22]. Our patients carrying the DD genotype had increased sBP (p 0.05). A similar finding was supported by other studies that the DD genotype was connected with higher blood pressure [23, 24].

On the other hand, homozygotes for the insertion have the lowest level of serum ACE and a better prognosis in terms of the development of DN [25, 26]. Several genetic-based studies showed that carriers of the ACE II genotype have an increased risk of diabetes in CVD patients [23, 27]. The ACE ID genotype is an independent factor influencing the development of cardiovascular complications and DN in patients with DM. Our patients carrying the ID genotype had a higher prevalence of CVD in family history (p 0.006). We would encourage careful monitoring of the BP and renal function of diabetic children who have CVD in their family history, especially patients with the DI genotype of the ACE gene.

In the group with MA, the majority of patients (62.5%) had the AA genotype of the AGTR1 gene, which is similar to the previous smaller study done on adolescents in the population of Serbia [15]. AA genotype was the most frequent in the non-MA group (49.1%) as well as in the total sample of patients (50.8%, 66 patients). It has been shown that people with the AA genotype have an increased risk of developing DN, while the CC genotype is associated with a lower risk of developing DN [28, 29]. Development of nephropathy is accelerated in the presence of hypertension and it is shown that the gene for AGTR1 may increase the risk of developing DN in patients with T1D through the effect on blood pressure. A large study showed that patients with T1D and hypertension had a more common AA genotype [28]. That is opposite to our result where patients with the AC genotype had a smaller nocturnal BP decline than patients with the AA genotype (P 0.025).

In the group with MA, the majority of patients (50%) had a CT genotype of the MTHFR gene. CT genotype was the most frequent in the non-MA group (45.6%) as well as in the total sample of patients (46.2%). Individuals homozygous for the T allele have reduced activity of MTHFR and elevated plasma homocysteine levels in comparison to those who are homozygous for the C allele. A large European study with adolescents with T1D showed that genotype CC is protective for the occurrence of MA [30]. Our patients with the CC genotype have statistically significant lower cystatin C values and a higher percentage of nocturnal decline compared to other genotypes.

The frequency of TT genotype 12.5% was similar to the finding by Smyth and al 12.2% [31] and Yuichiro et al 11% in patients with T1D [32]. They showed that rates of TT genotype were slightly greater in patients with DN. Also, patients carrying the CT genotype had higher triglyceride values. Our study didn’t show a connection between polymorphism in the MTHFR gene but we can be assumed that the CC genotype is protective for the occurrence of MA.

### Study limitations

The study is limited by the short duration of diabetes and the young age of the participants, which may influence the occurrence of DN and the non-significant association between the polymorphism of genes ACE, AGTR1, MTHFR, and DN. Some of our patients are likely to develop microalbuminuria over time, which highlights the need for extended follow-up and larger sample sizes in future studies.

## Conclusion

We showed a representation of different genotypes in adolescents with TID in a population of Serbia. In conclusion, our data suggest that common variants in the AGTR1, ACE, and MTHFR genes are not strongly associated with DN in our patients with T1D. The study was adequately powered to detect clinically relevant differences in the frequency of complications in carriers of different genotypes. Additional work is necessary to investigate the potential role of rare variants and epigenetic factors within these genes and others involved in the development of diabetic nephropathy in adolescents.

## Supporting information

S1 Table Raw data. (XLSX)
